# Late-Onset Group B Streptococcal Sepsis in Preterm Twins

**DOI:** 10.7759/cureus.38646

**Published:** 2023-05-06

**Authors:** Jubara Alallah, Khaild Ridnah, Bakur A Turkstani, Saeed N Albukhari

**Affiliations:** 1 Pediatrics/Neonatology, King Abdulaziz Medical City, Ministry of National Guard - Health Affairs, Jeddah, SAU; 2 Pediatrics, King Saud Bin Abdulaziz University for Health Sciences, Jeddah, SAU; 3 Neonatology, King Abdullah International Medical Research Center, Jeddah, SAU; 4 College of Medicine, King Saud Bin Abdulaziz University for Health Sciences, Jeddah, SAU; 5 Medicine, College of Medicine, King Saud Bin Abdulaziz University for Health Sciences, Jeddah, SAU

**Keywords:** nicu, twins, very low birth weight infant, late-onset sepsis, group b streptococcus

## Abstract

Group B streptococcal (GBS) infection is one of the leading causes of neonatal sepsis worldwide. Despite a significant decline in early-onset (EOS) sepsis due to intrapartum antibiotic prophylaxis, the incidence of late-onset (LOS) infection has remained unchanged. However, LOS GBS sepsis affecting twins is very rare. We report on preterm twins born at 29 weeks of gestation: Twin B was 31 days old when he developed LOS GBS sepsis and meningitis, and Twin A was 35 days old when he developed LOS GBS sepsis. Tests for maternal GBS colonization in breast milk were negative. Both babies were treated with antibiotics and eventually discharged without complications.

## Introduction

Group B streptococcal (GBS) infections in neonates are classified based on the time of onset. While early-onset (EOS) GBS infections develop shortly after birth (median age of 20 hours), late-onset (LOS) GBS infections develop between one week and 90 days after birth (mean age of three to four weeks). Each has a different clinical presentation and is treated accordingly. From 2006 to 2015, the incidence rate of EOS GBS infections decreased from 0.37 to 0.23 per 1,000 neonates due to the widespread use of intrapartum antibiotic prophylaxis (IAP); however, the incidence rate of LOS GBS infections remained the same (0.31 per 1,000 neonates) [1]. Of the six to seven million cases in 2012, approximately half a million resulted in death [2]. LOS GBS infections can cause various clinical conditions, including bacteremia, septic arthritis, osteomyelitis, and meningitis, and it is associated with a mortality rate of up to 10% and causes devastating neurologic consequences in 25-35% of survivors [3].

Neonatal GBS infections typically occur via transmission from the mother to the fetus during birth. GBS is a normal inhabitant of the genitourinary and gastrointestinal tracts; around 5-40% of women carry it as commensal bacteria. About half of these women’s newborns become colonized, but only 1-2% develop an infection. If the mother is colonized, predisposing factors such as infection, prematurity, preterm premature rupture of the membranes (PPROM) for more than 24 hours, maternal chorioamnionitis, and fever will increase the infant's susceptibility to infection. The risk of LOS GBS infections in cases of multiple gestations is higher if one sibling is affected [4]. In addition to vertical transmission from the mother, several studies have found that environmentally acquired GBS (e.g., from maternal breast milk) can cause LOS GBS infections [5,6].

We report on premature twins who developed LOS GBS infections with similar clinical features. Twin B was 31 days old when he developed LOS GBS sepsis and meningitis, and Twin A was 35 days old when he developed LOS GBS sepsis. Interestingly, Twin B’s blood culture on day 31 was positive but Twin A’s blood culture was negative. That is, Twin A was stable and had a negative inflammatory marker. Four days later, Twin A showed signs of sepsis; his blood culture was positive for GBS. Even though we suspected that maternal breast milk was the cause of enteral infection, two tests for GBS colonization in maternal breast milk were negative.

## Case presentation

The patients were male twins born to a 32-year-old mother (gravida 4 para 3) via cesarean delivery due to twin pregnancy, with no previous history of GBS colonization. Their gestational age was 29 weeks and four days, and both twins weighed 1400 g at birth. The mother was given one dose of dexamethasone and ampicillin antenatally. Antenatal urine samples and vaginal swabs were negative for GBS. Empirical antibiotic therapy with ampicillin and gentamicin was initiated in both infants because of suspected sepsis. Therapy was stopped 48 hours later, as blood cultures remained negative.

At delivery, Twin A’s Apgar score was 4 at one minute and 8 at five minutes. The baby required intubation and received surfactant replacement therapy. He was extubated in the NICU and required continuous positive airway pressure (CPAP) for seven days. A partial sepsis screen was done, and he was given ampicillin and gentamicin empirically for 48 hours. Antibiotics were discontinued when the cultures remained negative. Twin B had an Apgar score of 4 at one minute and 9 at five minutes. He was intubated and received surfactant replacement therapy. He was extubated in the NICU to a CPAP of 6 and FiO_2_ of 30% and was given a second dose of surfactant using the INSURE method. He required CPAP for five days. An initial partial sepsis screen was performed. He was given ampicillin and gentamicin for 48 hours. Antibiotics were discontinued when the cultures remained negative. Both babies did well with feeding protocol and routine NICU management.

At 31 days of age, Twin B began showing signs of illness; he was lethargic, exhibited significant bradycardia and desaturation, and had recurrent apnea. He was intubated and a full sepsis screen was performed. His blood culture showed GBS growth, as did his CSF culture (traumatic tap). The baby was treated for GBS meningitis. His initial CRP level increased from 28 to 119.5 mg/L. He required intubation for two days and nasal CPAP for an additional day. After that, his activity level improved and was considered clinically stable. He was treated with ampicillin for 14 days, as suggested by the pediatric infectious disease team. The GBS infection responded to ampicillin.

At 31 days of age, Twin A was asymptomatic and his blood culture was negative; no antibiotics were started. Four days later (at 35 days of age), he showed signs of lethargy and exhibited significant desaturation. A full sepsis screen was performed, and ampicillin and gentamicin were started. Blood culture revealed that the GBS was resistant to ampicillin, and hence it was substituted with cefotaxime. A blood culture taken three days later showed no growth. He received antibiotics for 10 days. His CSF culture was negative. Tests for GBS colonization in maternal breast milk were conducted twice and were negative both times.

Both babies were discharged in good condition with a normal clinical examination. Follow-ups at the neonatology clinic in the following year showed normal growth and development.

## Discussion

GBS is the most common pathogen that leads to neonatal sepsis, which is a major health problem among neonates worldwide [7]. Neonatal GBS infections can be divided into two categories: early-onset and late-onset. It has been shown that both the early- and late-onset types of the disease are identical clinically and microbiologically [8,9].

A meta-analysis conducted by Fleischmann-Struzek et al. [10], which included 1,270 studies published between 1979 and 2016, reported the incidence of sepsis in neonates and children globally. Neonatal sepsis was estimated to occur in 2,202 per 100,000 live births, and mortality was estimated to be between 11% and 19%. Madrid et al. [11] analyzed 135 studies in a meta-analysis and found that the pooled incidence of GBS infection in neonates was 0.49 (95% CI: 0.43-0.56) per 1,000 live births. The incidences of EOS and LOS GBS infections were 0.41 (95% CI: 0.36-0.47) and 0.26 (95% CI: 0.21-0.30), respectively. These results highlight the severity of neonatal GBS infection worldwide and identify it as a major health issue among neonates.

In most cases, neonatal sepsis occurs during the first 28 days of life [12]. However, an Irish study reported that about one-third of neonatal GBS infection cases involved LOS sepsis. Furthermore, they found that the fatality rate of LOS GBS infection was 7.7%, which was higher than the EOS fatality rate of 5.2% [13]. Giannoni et al. [14] studied 429 neonates to determine the differences between the incidence rates of EOS, hospital-acquired LOS, and community-acquired LOS GBS infections. They found 444 episodes of culture-proven sepsis, 20% of which were EOS, 62% were hospital-acquired LOS, and 18% were community-acquired LOS GBS infections. The researchers also found that the fatality rates of the EOS, hospital-acquired LOS, and community-acquired LOS GBS infections were 18%, 12%, and 0%, respectively. The most common microorganisms isolated in the cases were GBS, E. coli, and other Gram-positive bacteria.

Berardi et al. [15] assessed the possibility of GBS colonization in 160 mother-baby pairs. They took samples for culture from the milk, rectum, and vagina of the mothers to determine the source of infection. Samples were taken multiple times from the time of delivery until eight weeks postpartum. A total of 83 women and 30 neonates were found to be culture-positive. The mother-baby duos who were culture-positive showed the same GBS strain, which was confirmed by molecular typing. Therefore, the postpartum transmission of GBS from mother to baby is a viable cause of LOS neonatal sepsis.

Clinically, neonatal GBS sepsis presents with fever, difficulty feeding, irritability, lethargy, difficulty breathing, and cyanosis. If not detected early and promptly treated, it can lead to bacteremia and/or meningitis. Therefore, prior antenatal testing for GBS and the administration of antibiotics are needed to prevent such complications [16]. Empiric therapy using ampicillin and an aminoglycoside is the standard of care when neonatal GBS is suspected. The use of penicillin G monotherapy is recommended once GBS has been isolated in the culture. Dosage varies depending on the age of the patient. Infants up to seven days of age are given 250,000-450,000 units/kg/day, while infants aged seven days or older are given 450,000-500,000 units/kg/day. For uncomplicated bacteremia, patients are treated for 10 days. In the case of uncomplicated meningitis, patients are treated for 14 days. However, the treatment duration can be prolonged in complicated cases [17].

In this case, Twin A was given ampicillin empirically while awaiting his full septic screening results. The results showed resistance to ampicillin, and hence cefotaxime was used instead. Antibiotic therapy was continued for 10 days. Meningitis was excluded when the CSF screening results returned negative. Twin B was also given ampicillin empirically while awaiting full septic screening results. His clinical presentation raised suspicions of meningitis, and his CSF showed growth of GBS. In contrast to Twin A, Twin B’s culture results did not show resistance to ampicillin, which he was given for a total of 14 days. We cultured the mother’s milk to identify the source of the infection. However, the results were inconclusive. While the source of the infection has yet to be determined, this lack of information did not affect treatment options or duration.

This case study demonstrates that even when newborns pass the standard age range for contracting a GBS infection, they are not past the possibility of developing the disease. LOS GBS infections can present at any time within 90 days of birth. Moreover, a previously resolved infection does not exclude the possibility of another infection in the near future.

## Conclusions

Neonatal GBS infections are common but serious entities and can present with various clinical presentations at various times following delivery. Even though the disease generally presents early, it can also occur later and/or after another infection. If left untreated, neonatal GBS infections can lead to serious morbidity and mortality. Therefore, physicians must always be vigilant of the signs and symptoms of neonatal GBS infections, regardless of patients' age, especially when dealing with preterm infants.
